# Synthesis and performance evaluation of PES/chitosan membranes coated with polyamide for acid mine drainage treatment

**DOI:** 10.1038/s41598-019-53512-8

**Published:** 2019-11-27

**Authors:** Mathaba J. Machodi, Michael O. Daramola

**Affiliations:** School of Chemical and Metallurgical Engineering, Faculty of Engineering and the Built Environment, University of the Witwatersrand, Private Bag X3, Wits, 2050 Johannesburg South Africa

**Keywords:** Environmental sciences, Engineering

## Abstract

In this article, performance evaluation of PES membrane infused with chitosan and coated with polyamide layer for treatment of acid mine drainage (AMD) is reported. PES/chitosan membranes were fabricated by varying chitosan concentration (0, 0.5, 0.75 and 1 wt%) using phase inversion method. PES/chitosan membranes were coated with polyamide (PA) via co-solvent assisted interfacial polymerization technique (CAIP). Scanning electron microscopy (SEM) and contact angle analysis show that chitosan and polyamide could enhance permeability without affecting rejection of the membrane. The permeability was improved with increasing chitosan content. Atomic absorption spectroscopy (AAS) was used to quantitatively determine cations in the permeate and the sulphate ions were analysed using ultraviolet and visible (UV-VIS) spectrophotometer. Pure water flux of PES/PA membrane was significantly improved from 56 to 93 l/m^2^.hr with 1 wt% chitosan addition. Cation rejection (90.4, 88.3, 89.3 and 75.7% for Mn^2+^,Fe^2+^, Mg^2+^ and Ca^2+^, respectively) was observed to be higher than anion rejection (56.33% for SO_4_^2−^), when chitosan content was 0.75 wt%. These results indicate that the positively charged membranes under acidic condition had strong repulsive forces with the cations than attractions forces with anions. Polyethersulphone membrane modified with chitosan and coated with polyamide layer displayed potential for application in treatment of AMD.

## Introduction

Membrane technology application and research for treatment of wastewater has been growing rapidly due to global environmental concerns and the need for high quality water demand^1^. Amongst other polymeric membranes, polyethersulphone (PES) and polysulphone (PSf) have gained significant progress in acid mine drainage (AMD) treatment because of their high chemical and thermal resistance, mechanical stability in hot and wet conditions, and high permeability^2^. Although PES exhibits higher degree of hydrophilicity compared to PSf, its inherent hydrophobic character results in serious membrane fouling which leads to deterioration in permeation flux, shortened membrane lifespan and unpredictable separation efficiency^3^. The inevitable normal significance of semi-permeability and selectivity during membrane operation is the accumulation of particles and solutes on the membrane surface, within the matrix and porous structure^4^. The accumulated molecules form a layer on the surface of the membrane which hinders solvent movement across the membrane and generates osmotic back pressure which diminishes the effective transmembrane pressure (TMP) of the system^5^. Particulates, ions, macromolecules and biological substances are common foulants causing trouble during membrane operation. Organic matter is the most challenging and causes both reversible and irreversible fouling because it is common in natural water^6^. The relative resistance to cleaning is a distinguishing factor between reversible and irreversible fouling. Reversible fouling is the type that can be cleaned, and irreversible fouling remains even after cleaning. Irreversible fouling that remains after hydraulic cleaning technique is termed hydraulically irreversible fouling and that which remain after chemical cleaning is called chemically irreversible fouling^7^.

Several interventions have been used to increase the hydrophilicity of PES during wastewater treatment to avoid quick membrane replacement caused by irreversible surface fouling and internal fouling^8^. Pure polymeric and modified membranes that feature low fouling character and ability to restore water flux after cleaning would lower the replacement and maintenance cost of the technology during wastewater treatment. One approach to creating such membranes is blending and coating PES membranes with hydrophilic polymers^2^. The advantage of blending with hydrophilic polymers such as chitosan is that modification takes place even within the pores and not only on the membrane surface^9^. Chitosan, which is a biopolymer obtained through partial N-deacetylation of chitin, contains one primary amino and two free hydroxyl functional groups for each C_3_ and C_6_ building unit^10^. The reactive amino groups bind to virtually all group III transition metals but not group II and I. Under acidic medium, the amine groups on the chitosan’s structure get protonated and this leads to adsorption of anions through ion exchange. There is growing interest in membrane technology by scientists to use chitosan as raw membrane material or as an additive because of its non-toxic, biodegradable, biocompatible and antimicrobial properties^11^. Several studies have reported on membrane modification with chitosan to produce hydrophilic membranes with high antifouling property. The chitosan modified membranes exhibited effective surface with smaller pore sizes than unmodified membranes^12^. Another approach to improving antifouling property and surface characteristic of membranes is coating with hydrophilic polyamide layer. Polyamide offers numerous functional groups such as amines, free carboxylic acid and unreacted acylchloride group which are prone to modification and can act as binding sites^13,14^. Hydrophilic polymers containing polyamide and amines have been reported to be extremely effective in enhancing hydrophilic nature and selective properties of polymeric membranes.

Against this background, this study aimed to synthesis PES membrane blended with chitosan polymer and coated with polyamide layer for AMD treatment. The effect of chitosan addition and coating polyamide layer on surface morphology, hydrophilicity, permeability and selectivity of the membranes were investigated. Polyethersulphone provides polymer matrix while chitosan and polyamide were used as functional polymers.

## Results and Discussion

### Characterization of chitosan and membrane

**Figure 1:**
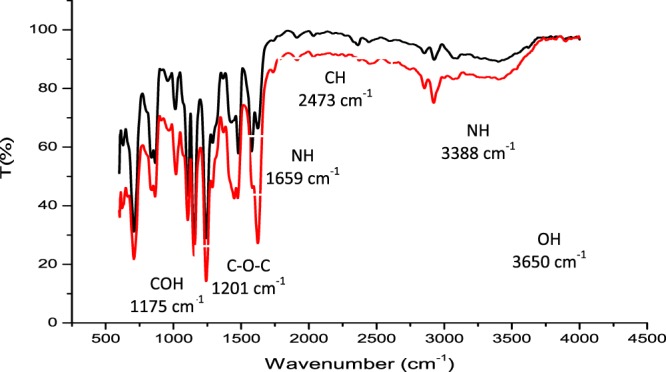
FTIR spectra of chitosan sample prepared in duplicate. Functional groups are assigned to the peaks and bands to indicate their location.

FTIR analysis was conducted on the synthesised chitosan sample to observe and verify functional groups present. Figure 1 presents the FTIR spectra of the chitosan sample. It was also used to determine the degree of deacetylation (DD) of the synthesised chitosan using Eqs (1) and (2). Duplicate samples were prepared, and as observed in Fig. 1, the samples yielded similar FTIR spectrum, indicating accuracy and repeatability of the synthesis process. The FTIR spectrum depicts typical amine peaks at around 3388 cm^-1^ and 1659 cm^−1^, with COH peak at 1175 cm^−1^, and the COC representative peak was identified at 1201 cm^−1^. CN peak was identified at 2927 cm^−1^ while the vibration at 2619 and 2473 cm^−1^ were assigned to CH. The degree of deacetylation of the chitosan samples was determined as 90.17% using Eqs (1) and (2). This indicates that enough amine groups have been exposed to act as potential contaminant binding sites.

**Figure 2:**
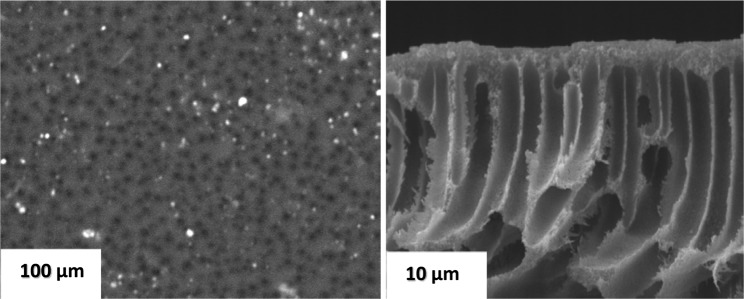
SEM images of surface and cross-sectional view of PES base support structure.

**Figure 3:**
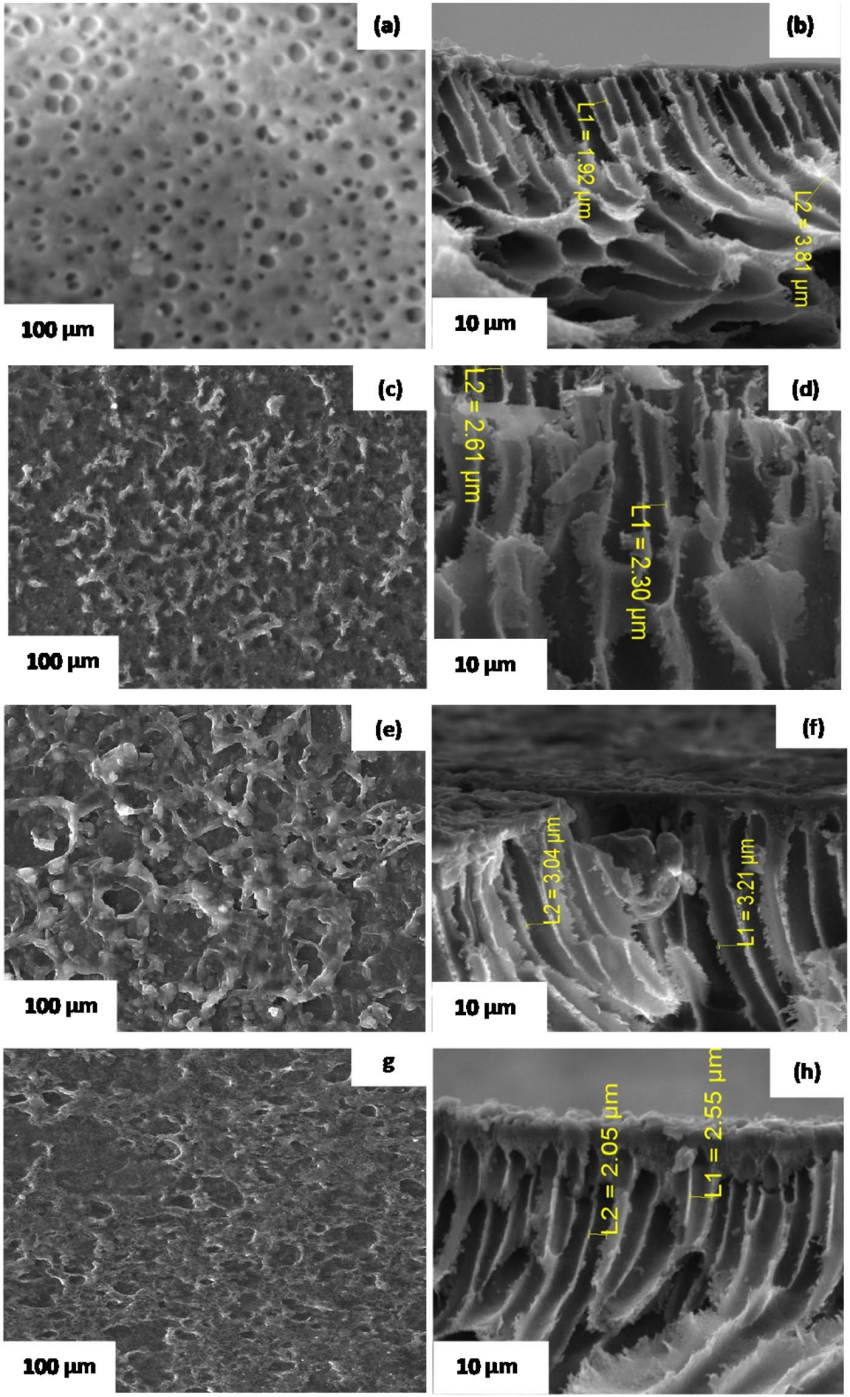
SEM micrographs and cross-sectional view of (**a**,**b**) PES/PA, (**c**,**d**) PES/0.5 wt% chitosan/PA, (**e**,**f**) PES/0.75 wt% chitosan/PA and (**g**,**h**) PES/1 wt% chitosan/PA membranes.

Surface morphology and cross-sectional view of pristine PES membrane are depicted in Fig. 2. Figure 2 shows a smooth and integrity surface structure with uniformly distributed pores. In comparison with PES membranes blended with chitosan and coated with polyamide layer shown in Fig. 3, there are obvious differences between surface morphology of polyamide layer formed over hydrophobic PES membrane (PES/PA) and those formed over PES membrane blended with hydrophilic chitosan (PES/0.5 wt% chitosan/PA, PES/0.75 wt%/PA and PES/1 wt%/PA). Ghosh and Hoek^15^ reported similar observation whereby the roughness character of the membrane reduced with increasing chitosan concentration probably due to chitosan forming complexes with PA structure and filling empty spaces of the PA layer. Moreover, typical surface morphologies of polyamide membranes prepared via conventional interfacial polymerization technique using piperazine and trimesoyl chloride with hexane alone as an organic solvent without acetone, depict a typical ridge- and – valley structure like that of commercial membranes^16,17^. However, co-solvent assisted interfacial polymerization was followed in this study, with acetone as a co-solvent. The surface morphology of PA membrane prepared by adding acetone as a co-solvent was greatly altered and the typical ridge – and – valley structure was flattened as compared to those prepared by conventional interfacial polymerization^18^. The L1 and L2 represents the underlying pore sizes of the membranes in the cross sectional view (Fig. 3(b,d,f,h). PES membranes are characterised with large pore size and coating polyamide material on the surface did not significantly reduce the underlying pore size. However, adding chitosan particles caused a reduction in pore size by occupying free space with 0.75 wt%, when compared with the largest pore size using 0.5 wt% and 1 wt%. Amine group on the chitosan structure reacts with the unreacted acylchloride group of polyamide via nucleophilic addition and/or elimination reaction. The reaction produces a large molecule which induced a stretch between pore which could increase the effective pore size of the membrane. No scientific clear trend was observed on the effect of chitosan concentration on pore size. It could be argued that at 0.75 wt%, enough amine groups were present to react with the unreacted acylchloride group. However, at 0.5 wt % not enough amine groups were introduced and at 1 wt% more than enough chitosan particles were introduced and started blocking more of the effective pore size.

**Figure 4:**
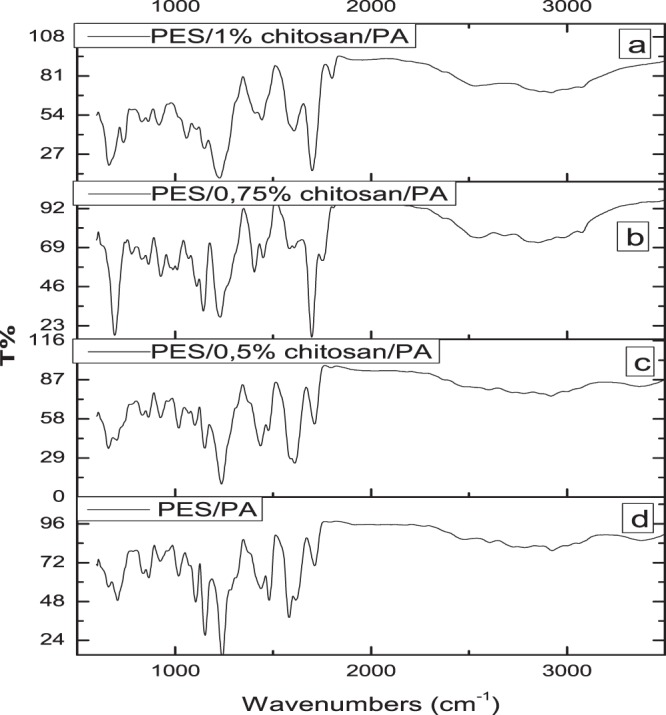
FTIR spectra of the synthesised PES membranes infused with chitosan and coated with polyamide layer. PES/PA membrane was not infused with chitosan.

The FTIR spectra of the membranes were collected and presented in Fig. 4 to obtain general information about the changes of PES membrane surface chemistry after blending and coating with chitosan and polyamide. As expected, all the prepared membranes showed typical characteristics of PES basic structure. The identified peaks at 660.46 cm^−1^, 660.16 cm^−1^, 691.87 cm^−1^ and 662.96 cm^−1^ was attributed to the C-stretching, and 866.02 cm^−1^, 864.54 cm^−1^, 864.96 cm^−1^ and 829.62 cm^−1^ to the C = C stretching on the aromatic ring structure for PES/PA, PES/0.5wt%/PA, PES/0.75wt%/PA and PES/1 wt%/PA membranes, respectively. The peaks at 1240.49 cm^−1^, 1237.03 cm^−1^, 1230.73 cm^−1^ and 1226.62 cm^−1^ are attributed to the sulfonyl (O=S=O) group while the aromatic ether (C-O-C) group is represented by the peak at 1153.21 cm^−1^, 1151.34 cm^−1^, 1144.34 cm^−1^ and 1149.81 cm^−1^ for PES/PA, PES/0.5 wt%/PA, PES/0.75 wt%/PA and PES/1 wt%/PA membranes, respectively. The strong band at 1712.23 cm^−1^, 1711.72 cm^−1^, 1752.98 cm^−1^ and 1700.01 cm^−1^ is associated with the stretching vibration of the C=O group of PES/PA, PES/0.5 wt%/PA, PES/0.75 wt%/PA and PES/1 wt%/PA membranes, respectively. The polyamide layer was coated onto the membrane using co-solvent assisted interfacial polymerization method instead of typical interfacial polymerization method. The broad band at around 3369 cm^−1^ was attributed to the N-H stretching frequency. In addition, the peak at 3380.91 cm^−1^, 3379.42 cm^−1^, 3072.30 cm^−1^ and 3075.46 cm^−1^ corresponded to the combined N-H stretching and C-N stretching vibrations for PES/PA, PES/0.5 wt%/PA, PES/0.75 wt%/PA and PES/1 wt%/PA membranes. The decreased intensity of C=O, N-H and combined N-H and C-N groups polyamide is reflective of the thin layer of polyamide produced via co-solvent assisted interfacial polymerization. CAIP makes it possible to develop a novel polymerization procedure which will effectively have control on the thickness of the polyamide dense layer and size of the nanopore. The advantage of this technique is that a thin miscible zone will be formed in the water/hexane and acetone system once the acetone content is controlled. Immiscible binary system of water and hexane will form and a wide liquid to liquid region for the ternary mixture will remain giving control of the interfacial polymerization process. Similar observations were reported in literature, whereby no significant difference was made between polyethersulphone and polyamide membranes^19,20^.

**Figure 5:**
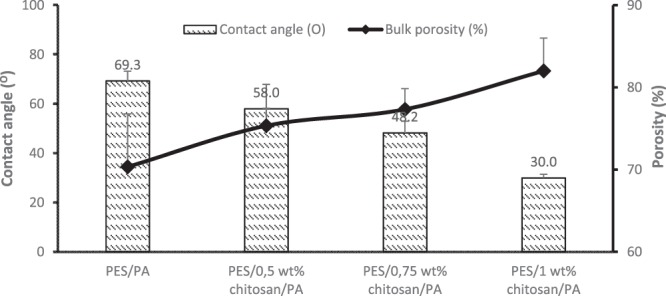
Static contact angle and bulk porosity measurement of the fabricated membranes.

Figure 5 shows overall porosity and contact angle of the prepared membranes. All the membranes blended with chitosan (PES/0.5 wt%/PA, PES/0.75 wt%/PA and PES/1 wt%/PA) showed an enhanced porosity and improved degree of hydrophilicity. Addition of chitosan particles influenced the wettability of the membranes (PES/0.5 wt%/PA, PES/0.75 wt%/PA and PES/1 wt%/PA) as compared to unblended PES/PA membrane. The contact angle was reduced by 39%, 18% and 9% for PES/1 wt%/PA, PES/0.75 wt%/PA and PES/0.5 wt%/PA, respectively. This decrease in contact angle with increasing chitosan content affirms the influence of chitosan as a hydrophilic agent to enhance the membrane surface hydrophilicity. Introduction of chitosan improved the degree of hydrophilicity of the fabricated membranes by 58%. The addition of chitosan supported hydrophilic amide sites introduced by coating with polyamide layer which resulted in enhancing transport of water molecules through the membrane.

### Performance evaluation of the membranes for AMD treatment

**Figure 6:**
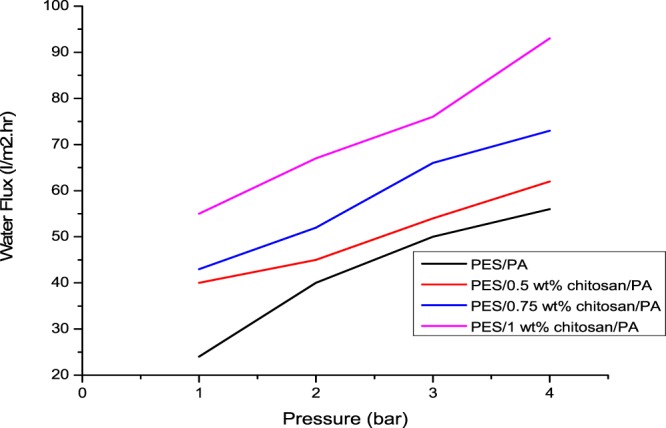
Pure water flux of the synthesised membranes against operating pressure.

**Figure 7:**
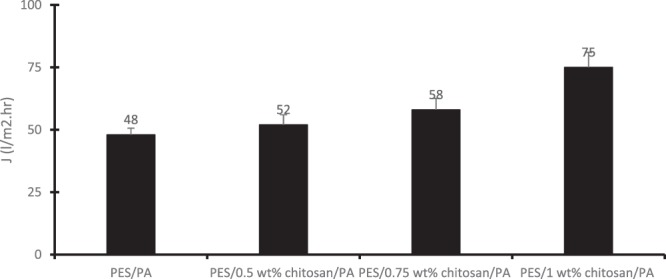
Flux measured by permeating AMD solution through the membranes at 4 bar.

Pure water flux and permeate flux of the membranes were measured as a function of pressure (Figs. 6 and 7). All four membranes had a linear volumetric water flux increase with increasing pressure from 1 to 4 bar. Polyamide materials are usually used to construct reverse osmosis membranes, which have smaller pore size compared to other membranes, hence the observed low water flux. Effective membrane thickness and pore size also influence solute/solvent permeability through the membrane (Shockravi *et al*., 2017). Although the PA layer contributed to the reduced water permeability, increasing chitosan content improved the water flux from 56 l/m^2^h to 62 l/m^2^h, 73 l/m^2^h and 93 l/m^2^h for PES/0.5 wt%/PA, PES/0.75 wt%/PA and PES/1 wt% /PA, respectively. This could be attributed to the fact that the interaction of chitosan’s amine group and PA active layer’s unreacted acylchloride group created a thin layer on the membrane surface. Additional chitosan’s amine groups which could not interact with unreacted acylchloride groups favoured sorption of water molecules by the membrane^21^. This phenomenon led to improved permeate flux of 93 l/m^2^h for PES/1 wt% /PA compared to 73 l/m^2^h of PES/0.75 wt%/PA. Similar observations were made in literature^18^. The permeability of the membranes was 14 l/m^2^h, 15.5 l/m^2^h, 18.25 l/m^2^h and 23.25 l/m^2^h.bar for PES/PA, PES/0.5 wt%/PA, PES/0.75 wt%/PA and PES/1 wt%/PA, respectively.

Figure 7 shows the permeate flux of AMD solution through the membranes having different chitosan content. The feed AMD solution used in this study had a pH of 3.2. Charge density of a membrane depends on various functional groups on its surface, chemical structure of the membrane due to dissociation of the functional groups and migration of charged solutes from solution onto the membrane surface^18,22^. Coating polyamide layer onto the PES/chitosan membrane to produce PES/chitosan/PA membrane provided amide, amines, carboxylic and alcoholic functional groups onto the membrane surface. Chitosan blended within PES granules will provide amine groups inside the membrane matrix. During membrane rejection process, partial hydrolysis of polyamide leads to formation of ammonium (-NH_3_^+^) and carboxyl (-COOH) groups. At low pH (±3.2) carboxyl groups are undissociated and the amine groups are protonated to form quaternary amine groups, and the membrane becomes positively charged while the carboxyl groups are dissociated, and the membranes become negatively charged. As reported in literature, high permeate flux rate is associated with strong forces on the membrane surface wall which prevents accumulation of ions and formation of gel layer on the membrane surface^23^. This behaviour is best described by Childress *et al*.^24^. The authors argued that the behaviour can be determined by several mechanisms such as membrane size modification, osmotic pressure gradient and electro-viscous effect. When membrane is charged, the charged groups on the membrane material assume an extended configuration due to electrostatic repulsive forces between them (like charges repel each other). Thus, it causes the membrane pore size to diminish and subsequently decreases permeate flux and increases rejection. In this study, it was observed that with increasing chitosan content, the permeate flux was increased. This could be attributed to the enhanced hydrophilic property of the membranes.

**Figure 8:**
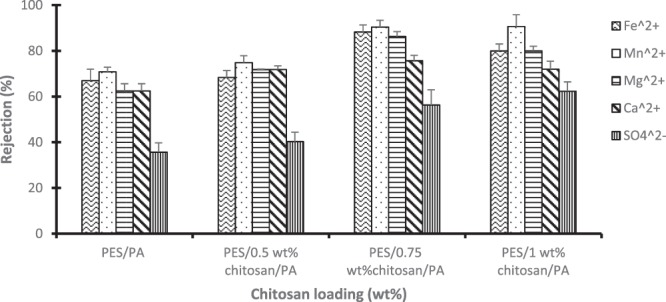
Rejection (%) of metal and sulphates ions using PES/chitosan membranes coated with polyamide layer having various chitosan loading.

Figure 8 presents rejection of selected metal and sulphates ions by PES/PA and PES/PA blended with chitosan biopolymer membranes. Although the addition of chitosan in the PES/PA blend had increasing linear effect on the membrane permeability, such could not be reported on metal and sulphate ion rejection. All membranes had increased rejection for all selected contaminants until when chitosan content was 0.75 wt%. At this loading, highest rejection of 88.3%, 90.4%, 89.3%, 75.7% and 56.33%, was observed for Fe^2+^, Mn^2+^, Mg^2+^, Ca^2+^, and SO_4_^2+^, respectively. Further chitosan addition either had no significant on rejection or induced a decline. The electrostatic viscous effect is observed when an electrolyte passes through a charged surface pore. The AMD was at pH of 3.2, meaning the membrane was positively charged due to protonation of the amine groups. The osmotic pressure near the membrane surface increases at low pH. This is attributed to retention of sulphate ions which interacts with the positively charged membranes and the electrostatic repulsive forces generated between cations and the membranes surface^25^. As reported in Figs. 6 and 8, the porosity and water flux of the membranes was enhanced by chitosan addition due to the improved hydrophilic nature of the membranes. The declining contaminant rejection could be attributed to the fact that at high fluxes, metal and sulphate ions retained on the membrane surface were pushed through the membranes into the filtrate stream. For PES/PA membrane which had no chitosan in its blend, 67%, 70.8%, 78.9%, 59.3% and 62.5% rejection were achieved for Fe^2+^, Mn^2+^, Mg^2+^, Ca^2+^ and SO_4_^2−^, respectively. The results are better than what was reported by Mthethwa^26^ using PES membrane to treat AMD without modification. PES/0.5 wt% chitosan/PA, PES/0.75 wt% chitosan/PA and PES/1 wt% chitosan/PA had significant metal and sulphate ion rejection increase compared to PES/PA. This was due to introduction of more amine functional groups which when protonated repel the cations or attract anions.

**Figure 9:**
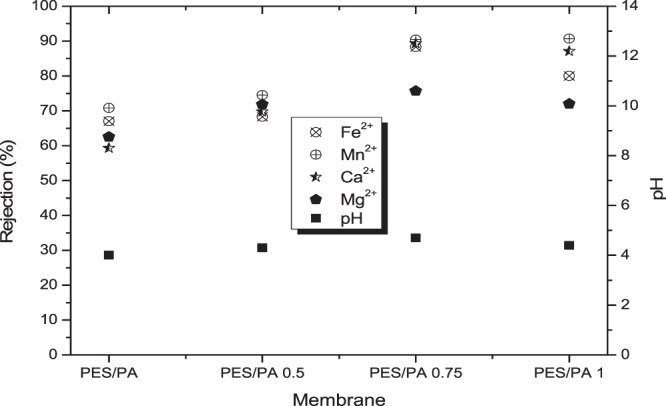
Rejection of selected cations by the membranes and the measured pH.

Literature argues that metal ions tend to form metal complexes with OH^−^ groups at higher pH and membrane rejection favour metal complexes than metal ions^27^. As depicted in Fig. 9, the pH was acidic (4 ± 0.7) throughout, therefore it can be deduced that the cations were removed as metal ions. Under acidic conditions, the amine and amide functional groups on the chitosan and polyamide structures get positively charged^28^. This caused the membrane to be positively charged. Rejection of anions takes advantage of appositional charges between the positively charged membrane and anions through electrostatic attraction force, however, the low anion removal shows that repulsion force were stronger than attraction force. In addition, small cations have high mobility compared to the large sulphate ions.

It is worth stating that the influence of pH on the membrane flux and ion rejection was not investigated in the study, however, the pH of the permeate was reported and is presented in Fig. 9. The pH of the synthetic AMD was 3.2., and Fig. 9 shows the permeate pH to be slightly higher. Polymeric membranes tend to be positively and negatively charged at lower and higher pH, respectively^27^. This could be articulated in accord with sorption of water molecules through the membrane. Sorption of water molecules from acid solution through a membrane triggers a slight pH shift towards neutrality. Considering the rejection of cations (Mn^2+^, Fe^2+^, Mg^2+^ and Ca^2+^), it is worth mentioning that the metals were treated simultaneously, not separately. Therefore, the interaction amongst the metals can influence the rejection efficiency. Hydroxide of Mn^2+^, Fe^2+^, Mg^2+^ and Ca^2+^ is reported in literature to precipitate at pH of 9.0 to 9.5, 5.5 to 8.5, >9 and >10, respectively^29,30^.

The pH was noted to not change significantly for PES/PA which had no chitosan and for PES/0.5 wt% chitosan/PA membranes. However, for PES/0.75 wt%/PA and PES/1 wt%/PA membranes the pH increased to 4.3. This behaviour could be attributed to the addition OH^−^ groups available on the chitosan structure. Fe^2+^ reported high rejection compared to other metal complexes and this could be attributed to the fact that it started to form hydroxide at lower pH of 5.5. It was followed by Mn^2+^ due to the fact that Mn^2+^ will simultaneously precipitate with Fe^2+^ at pH below 8, provided the concentration of Fe^2+^ is much greater than that of Mn^2+^^29^. Table 1 gives an overview of some comparative studies reported in literature. The flux achieved in this study was significantly higher than the fluxes of most studies reported in literature and the rejection of contaminants improved too.

**Table 1:** Performance of membranes in this study compared with other studies in literature.

Membrane	Target contaminant	Outcomes	Reference
Commercial CPA2, ESPA1 Polyamide membranes	Stainless steel wastewater (Cr, Ni, Fe, Mn, Cu, Zn, NO_3_-N, NO_2_ but the target was NO_3_-N)	1. ESPA1 had a flux of ± 39 l/m^2^.hr and CPA2 had ± 18 l/m^2^.hr 2. Rejection of polyamide membranes was between 90 to 99% for 1000 to 60 mg/L of NO_3_-N	Kim *et al*.^36^
PES/PA membrane	BSA	1. Unblended PES had 7.5 l/m^2^.hr and PES blended with 2 wt% PA had 80.4 l/m^2^.hr	Shockravi *et al*.^2^
Commercial AFC NF polyamide membrane	Pb(NO_3_)_2_	1. Flux of ±30 l/m^2^.hr 2. Reported rejection of 99.4% for 50 mg/L of Pb(NO_3_)_2_ at pH of 5.7	Gherasim *et al*.^22^
PA/chitosan membrane	NaCl, CaCl_2_ and Na_2_SO_4_	1. Flux was between 32.9 to 59.6 l/m^2^.hr 2. Rejection of ±38% for NaCl, CaCl_2_ at 93.8% and 97.3% for Na_2_SO_4_	Akbari *et al*.^18^
PES/chitosan/PA membrane	Fe^2+^, Ca^2+^, Mn^2+^, Mg^2+^, and SO_4_^2−^	1. Maximum flux of 93 l/m^2^.hr 2. Cation rejection (90.4% Mn^2+^,88.3 Fe^2+^, 86.3%, % Mg^2+^ and 75.7% Ca^2+^) and 56.33% for SO_4_^2−^	This study

**Figure 10:**
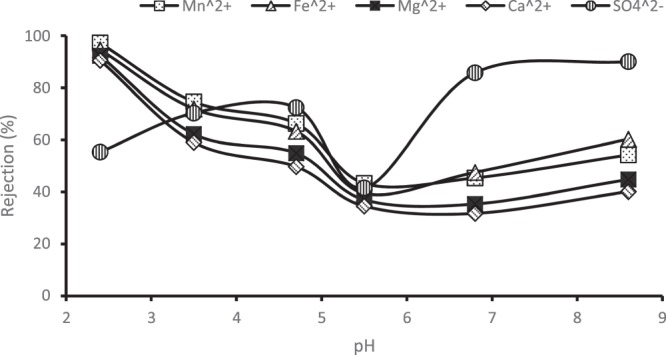
Effect of pH on rejection efficiency of PES/0.75 wt%/PA membrane.

Figure 10 depicts the effect of feed solution pH on the rejection efficiency of PES membrane infused 0.75 wt% chitosan and coated with polyamide layer. The rejection of the ions decreased to its lowest at pH of around 5.7, but increased again when pH increased. This behaviour could be attributed to the isoelectric point (IEP) or zero potential charge effect within a certain pH range of the membrane. Charge density of a membrane depends on various functional groups on its surface, chemical structure of the membrane due to dissociation of the functional groups, and migration of charged solutes from solution on to the membrane surface^18,22^. Literature has shown that pH corresponding to a peak in flux reporting the lowest rejection of ions by a membrane indicates the IEP or zero potential charge of the membrane^26^. Lowest rejection and peak in flux for both PES/chitosan and PES/chitosan/PA membranes are reported at pH between 5 and 6. In comparison with literature, Mthethwa^26^ reported IEP of PES membrane to be around pH of 5.04 during the treatment of acid mine drainage. Gherasim *et al*.^22^ observed the IEP of an AFC 80 Nanofiltration membrane to occur at a pH of about 5.7 when exposed to Pb(NO_3_)_2_ solution. AFC 80 is a thin-film composite membrane consisting of a polyamide skin-layer on top of polysulphone support supplied by PCI membrane systems. It can further be concluded that the surface charge of the membrane becomes positive at a pH lower than 5.5 but negative at a pH above 5.5.

### Membrane performance evaluation using real industrial AMD

**Figure 11:**
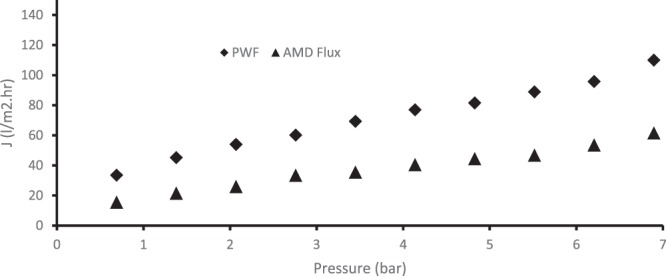
Pure water and Industrial AMD flux through the membrane using a cross flow filtration system.

It is important to note that membrane infused with 0.75 wt% chitosan was selected, based on the results from the use of synthetic AMD, and then used for real AMD treatment. The composition of the real AMD is presented in Table 3. The pH of the real AMD was 2.7. Figure 11 depicts the flux of pure water and that of the industrial AMD through the membrane. The flux of the membrane for both pure water and AMD solution was established by noting down the time taken to collect 250 mL of the permeate volume. High pure water flux of 110 l/m^2^.h was obtained when the pressure was 6.9 bar while flux of AMD through the membrane at the same pressure was 61.58 l/m^2^.h. The reduction in the membrane flux during real AMD treatment is expected when compared to the pure water flux because the real AMD contains contaminants which could lead to concentration polarisation on the membrane surface.

**Figure 12:**
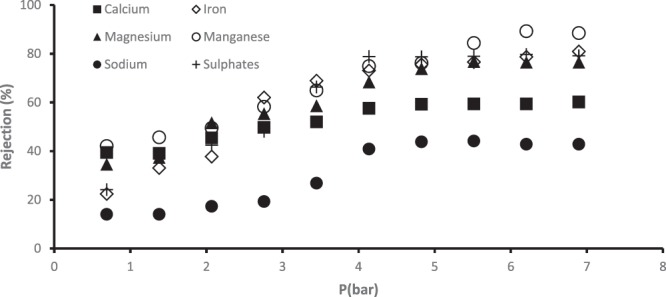
Rejection of selected components from the real AMD by PES/chitosan/PA membrane.

Figure 12 shows the rejection of metal and sulphate ions. Although the real AMD consists of 2.1 mg/L of Ni^2+^, but no nickel was detected in the permeate. This could be attributed to the very low concentration of this ion in the AMD and it could be that the membrane displayed 100% rejection of the nickel during the treatment. Rejection of both metal and sulphate ions improved with increased pressure, with Ca^2+^, Fe^2+^, Mg^2+^, Mg^2+^, Na^+^ and SO_4_^2−^ reaching a maximum rejection of 60.14, 80.89, 76.46, 88.46, 42.81 and 79.13%, respectively. Rejection of Na^+^ was poor and this behaviour could be attributed to the particle size exclusion theory, that is, membranes remove divalent ions more than monovalent.

Infusing chitosan within the PES membrane and coating it with polyamide layer on top introduced function groups such as OH^−^, -NH_2_, NH_3_^+^ and -COOH mainly. The cation removal mechanism could be attributed to the strong repulsive forces formed between the positively charged membrane due to the protonation of the chitosan amine groups and the cations. In acidic solutions, the amine groups on the chitosan structure attract proton to form quaternary amine groups which consequently gives the membrane more positive charges. Sulphate removal could be attributed to the sieving mechanism and electrostatic attraction force generated by the positively charged membrane and the anions. Comparing the rejection of the cations (Mn^2+^, Fe^2+^, Mg^2+^, Na^+^ Ca^2+^), it is important to mention that the metals were treated as a mixture, not as a single component. Therefore, the interaction amongst the metals can influence the rejection efficiency.

### Fouling resistance of the membrane

**Figure 13:**
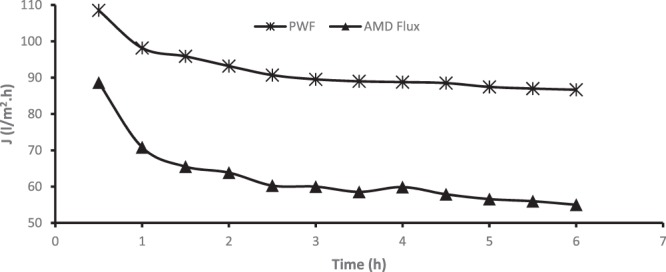
Permeate flux of pure water and AMD as a function of time.

Figure 13 shows the flux of pure water and that of the real industrial AMD through the membrane from 0 h to 6 h at a step increase of 1 h. A reduction of about 20 l/m^2^.h in the membrane flux during pure water permeation and of about 40 l/m^2^.h during the AMD treatment, as shown in Fig. 13, could be attributed to fouling or concentration polarization of the membrane. However, considering membrane flux during the AMD treatment, a sharp decrease in membrane flux could be observed in the first 30 minutes and a drastic decline of the flux continued for about 3 hours. This loss in flux is attributed to the concentration polarisation which forms a layer on the membrane surface and obstructs solvent movement through the membrane.

**Figure 14:**
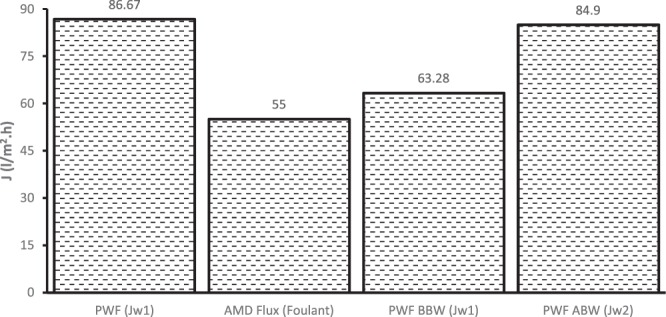
Recovery of membrane fouling after AMD treatment.

To understand the degree of fouling and recover the flux of the fouled membrane after 6-hour operation, the fouled membrane was backwashed by permeating pure water through the membrane. Figure 14 shows the results of initial membrane flux (PWF $$({J}_{w1})$$ and membrane flux after AMD treatment ($${J}_{AMD})$$, membrane flux of pure water permeation before backwashing ($$PWF\,BBW)$$ and membrane flux during pure water permeation after backwashing $$(PWF\,ABW)$$. Equation (7) was used to determine the reversible resistance of the membrane and it was established that 44% of reversible resistance was observed by permeation of AMD solution through the membranes after 6 hours at 6.9 bars. The higher percentage of reversible resistance (sometimes referred to as cake resistance) could be attributed to the concentration polarisation. However, real industrial AMD contains mixture of organic and inorganic compounds which could accelerate concentration polarisation, and subsequently fouling. After AMD treatment, membrane flux was 63.28 l/m^2^.hr, which indicates a 27% loss of the original membrane flux. Backwashing was performed and the membrane flux after backwashing was 84.9 l/m^2^.hr, indicating a 2% loss of the original membrane flux after backwashing of the AMD-fouled membrane. Even the flux recovery ratio (FRR) of 97.96%, obtained using Eq. (6), corroborates this observation. The 2% loss of the membrane flux after backwashing could be regarded as irreversible resistance, obtained from Eq. (8), which could not be removed by hydraulic backwashing. This observation infers that the membrane (PES/chitosan/PA membranes) developed and tested in this study possesses excellent antifouling properties.

## Conclusion

According to this study, PES membrane was modified by blending PES membrane with chitosan and coating with polyamide layer using co-solvent assisted interfacial polymerization. The SEM images show membranes with smooth surface roughness to those prepared by conventional interfacial polymerization. Additionally, polyamide layer formed on hydrophilic structures is smoother than those formed on hydrophobic structure. These hydrophilic polymers (chitosan and polyamide) could enhance the hydrophilic property of PES membrane without affecting rejection. Addition of these co-polymers induced a downward trend in contact angle of the membranes signifying improved hydrophilic property. The prepared membranes showed enhanced porosity and permeability with increasing chitosan created by the interaction of chitosan’s amine and unreacted acylchloride of the polyamide polymer. Since metal ions form complexes with OH^−^ at higher pH, it could be concluded that the metal ions in this study were removed as metal ions since the observed pH was acidic throughout. High metal and sulphate ions rejection was observed when chitosan content was 0.75 wt%. Further addition up to 1 wt% did not have significant effect on rejection or it caused a decline although permeability was the highest compared to other membranes. PES membranes blended with chitosan and coated with polyamide layer have great potential to remove metal and sulphate ions from AMD.

## Methods

### Materials and chemicals

Sodium hydroxide (NaOH), Sulphuric acid (H_2_SO_4_), Hexane (C_6_H_14_), Acetone (C_3_H_6_O) and metal sulphates salts were purchased from Rochelle Chemicals & Lab Equipment. Piperazine (PIP), Triethylamine(TEA), Trimesoyl chloride(TMC), Dimethyl Sulfoxide (DMSO), and transparent polyethersulphone (PES) beads (3 mm) with molecular weight 58 000 g/mol were purchased from Sigma-Aldrich (Pty) Ltd, South Africa. No further purification was done as the chemicals were supplied as analytical grade. Sea shells collected from Durban South Beach, Rutherford were used to synthesis chitosan. The pH and conductivity were measured using Metler Toledo dual meter (Sevenduo pH/conductivity meter with a Metler Toledo inLab Pro ISM pH electrode and inLab 738 ISM conductivity probe).

### Synthetic AMD

Synthetic feed solution was prepared as per the characterized data obtained from Tutu *et al*.^31^, which is a composition of mine water collected from Randfontein(Black Reef Incline, 17 and 18 Winzes), Johannesburg in South African.Synthetic feed AMD solution was used to prevent competition of desired and undesired species present in real AMD. An appropriate amount of metal sulphate salts (Table 2) was dissolved in 1000 ml of deionized water and agitated for 30 minutes at 200 rpm to ensure complete dissolution and the pH was adjusted to 3.2 using 0.1 M Sulphuric acid and Sodium Hydroxide. The AMD solution was prepared and used on the same day without storage to ensure consistent quality.

**Table 2:** Synthetic AMD composition.

Salt dissolved	Species	Concentration (mg/L)
Na_2_SO_4_	SO_4_^2−^	4556
FeSO_4._7H_2_O	Fe^2+^	933
CaSO_4_.2H_2_O	Ca^2+^	461
MgSO_4_.7H_2_O	Mg^2+^	345
MnSO_4_.H_2_O	Mn^2+^	321

### Production of chitosan and synthesis of membrane

#### Chitosan production from chitin (sea shells)

Seashells were boiled in water (94 ± 5 °C) for 2 hours and dried in an oven at 120 °C for 1 hour. They were crushed and milled using milling pot and rods for 3 hours into fine powder. The steps deproteinization, demineralization and deacetylation were sequentially carried out to obtain chitosan from chitin as reported by Abdou *et al*.^32^

(i) Deproteinization
  Chitin was treated with 6% NaOH solution at 60 °C in a 500 mL Erlenmeyer flask. The mixture was kept under constant stirring for 2 hours on a heating plate equipped with a magnetic stirrer. After 2 hours, the mixture was kept undisturbed under ambient conditions to settle the chitin particles and the supernatant alkaline solution was decanted. The residual was washed with deionized water until neutral pH.
(ii) Demineralization
  The deproteinized chitin was subjected to a 6% HCl solution for 2 hours at 60 °C in a 500 mL Erlenmeyer flask. The mixture was kept under constant stirring for 2 hours on a heating plate equipped with a magnetic stirrer. After 2 hours, the mixture was kept undisturbed under ambient conditions to settle the chitin particles and the supernatant acidic solution was decanted. The residual was washed with deionized water until neutral pH.
(iii) Deacetylation

The deproteinized and demineralized chitin underwent treatment with 40% NaOH at 120 °C for 6 hours on a heating plate equipped with a magnetic stirrer.

Various methods are available in literature for chitosan characterisation, but the most discussed due to its simplicity is the infrared spectroscopy^33^. It is for this reason that FTIR was employed to characterize chitosan and to determine its degree of deacetylation (DD). The absorption band ration of A_1320_/A_1420_ have proven to show superior agreement between the absolute and estimated DD values^32,34^. The DD of the chitosan samples was determined using Eqs (1) & (2):

1 $${\rm{DA}} \% =13.9(\frac{{\rm{A1320}}}{{\rm{A1420}}})-12.20$$

2 $${\rm{DD}} \% =1-{\rm{DA}}$$

where DA% is percentage degree of acetylation and DD% is the degree of deacetylation

Duplicate chitosan samples were prepared, and average values were taken.

#### PES support and PES/chitosan membrane preparation

Polymeric solutions were prepared by dissolving the correct amount of PES beads (10 wt%) in Dimethyl Sulfoxide on a magnetic stirrer at room temperature measured as 24.8 °C on the day. Specific chitosan particles were added at different concentrations (0, 0.5, 0.75 and 1 wt%) after complete dissolution of PES granules. The casting solutions were agitated for 24 hours to obtain a homogenous gel. Dope solution was cast on a glass plate with a casting knife with a thickness set at 250 µm. After spreading the gel, the entire glass was immersed into a room temperature bath containing deionized water for 10 minutes. The membrane sheet was separated from the glass plate. The sheets were placed in deionized water for 24 hours to allow complete desorption of the solvent from the membrane. Finally, the membranes were dried in oven to evaporate any trapped water and/or solvent from the membrane at 60 °C. Digital Micrometer was used to measure the thickness of the membranes.

#### Polyamide layer formation

Co-solvent assisted interfacial polymerization^35^ technique was used to coat polyamide layer onto the PES and PES/chitosan membranes. Diamine aqueous solution was prepared by mixing 2 wt% of piperazine and 0.6 wt% triethylamine. Previously prepared PES and PES/ chitosan membranes were both dipped in the diamine solution for 120 s and placed between two filter papers to absorb any excess amine solution. The amine-saturated PES and PES/chitosan membranes were immediately immersed in an organic phase solution containing 0.1 wt% trimesoyl chloride and 5 wt% acetone in n*-*hexane for 60 seconds for conventional interfacial polymerization to occur. The resulting membrane were cured at 80 °C for 5 minutes in an oven.

### Characterization of chitosan and membranes

Surface morphology and cross-sectional view of the fabricated membranes was observed with Scanning Electron Microscopy (SEM), (TESCAN Vega 3xmu) equipped with EDS (OXFORD Xmas). Samples to be analysed with SEM should be conductive or semi conductive. However, polymeric materials and membrane are nonconductive by nature hence coating is necessary^28^. Samples for both surface morphology and cross section were exposed to carbon coating before mounting onto the specimen holder of the SEM machine. Additionally, samples for cross section were immersed in liquid nitrogen for 10 minutes and cryogenically fractured quickly by hand before carbon coating and mounting onto the specimen holder. The characteristic functional peaks of the produced chitosan particles and surface chemical structure of the membranes was analysed using Fourier Transform Infrared Spectroscopy (FTIR). The infrared spectra were recorded at room temperature in the wavenumber range of 4000 to 650 cm^−1^ using Perkin Elmer Spectrum. Particle size distribution of the synthesised chitosan was analysed using laser diffraction technique (Malvern Mastersizer 2000 instrument). The membrane’s degree of hydrophilicity was investigated using Dataphysics Optical contact angle analyser (OCA 15 EC GOP). The contact angles of de-ionized water were measured using the sessile drop method on a dried surface of the membranes. Ten measurements were taken and averaged on different locations of the membranes.

Bulk porosity of the membranes was estimated gravimetrically. Three pieces of membranes were cut and immersed in 2-propanol water for 24 hours at room temperature. Then the wet membranes were taken and placed between two filter papers to remove remaining solvent on the membrane surface and weighed to achieve wet weight (W_w_). Thereafter, the wet membranes were dried in oven at 50 °C for 2 hours and weighed to obtained dry weight (W_d_). The bulk porosity was obtained using Eq. (3):

3 $$Porosity\,( \% )=\frac{{W}_{w}-{W}_{d}}{A\times l\times {d}_{P}}\times 100$$

where A, is the membrane effective areas, *l* is the average thickness of the membranes measured using a digital Micrometer, *d*_*p*_ is the density of 2-propanol (0.786 g/cm^3^).

### Membrane permselectivity

The experiments were conducted on laboratory-scale dead-end filtration cell mainly consisting of a holding cell with a volume of 300 mL volume and effective filtration area of 14.6 cm^2^. The feed pressure was achieved by applying nitrogen gas. After the membrane was fixed, deionized water was passed through the membrane to pre-press and compact the membrane to ensure immersion of water. Pure water flux (J, L/m^2^ h) was determined at ambient temperature by permeating deionized water through the membrane. This was necessary to determine the initial flux of the membrane before evaluating with AMD. The water flux was estimated using Eq. (4):

4 $$PWF=\frac{V}{At}$$

where V (Liters) is the volume of permeated water, A (m^2^) is the effective membrane area and t (hours) is the filtration time, respectively. Membrane permeability was obtained by dividing pure water flux against the pressure applied (∆P). Synthetic AMD solution was fed through the membranes pressured with nitrogen gas and the filtrate was collected and analysed for metal ion content using Atomic Absorption Spectroscopy (Thermo scientific ICE 3000 series).

Sulphates were analysed using UV-VIS spectrophotomer by following the Environmental Protection Agency (EPA) method 3754. Filtrates for sulphates analysis were conditioned. The conditioning solution was prepared by mixing 100 ml 95% ethanol, 30 ml of HCl with 75 g NaCl in a 500 ml flask. Thereafter, glycerol was added to the mixture. Then 1 ml of the filtrates for sulphate analysis and 5 ml of the conditioning agent were transferred to another 500 ml flask and stirred on a magnetic stirrer. A spoonful BaCl_2_ was added and continued stirring for additional 5 minutes. Immediately after stirring the solution was placed into a cuvette to measure the turbidity of the solution for 4 minutes at 30 seconds intervals. A calibration curve was prepared by appropriate dilution of 100 ppm Na_2_SO_4_ bulk solution.

Rejection was determined using Eq. (5):

5 $$R=\frac{{C}_{f}-{C}_{p}}{{C}_{f}}\times \frac{100}{1} \% $$

where R is the percentage rejection, C_f_ and C_p_ (mg/L) are feed concentration and permeate concentration, respectively.

Synthesised membrane was evaluated using real industrial AMD to assess the performance and fouling potential using a cross flow filtration system. Industrial AMD was collected in Randfontein using polypropylene bottle (duplicate samples were collected using 5 Liter bottles). At first the sampling bottles were rinsed with the site AMD to ensure consistency between the sampling bottles and the sampling environment. The AMD was transported as it is without preservation because the distance between the sampling point and the laboratory were analysis were carried out was around 40 km reach. So therefore, no oxidation or interference which would change the quality of the AMD was expected. Upon arrival in the laboratory, the AMD samples were filtered using 0.45 µm filter paper before analysis were carried out. This was necessary to remove any suspended solids. The metal content was analysed using AAS and sulphates using UV-VIS. Table 3 shows the concentration of the metal and sulphate ions characterized in the industrial AMD. pH of the AMD was measured immediately after collection on site as 2.7.

**Table 3:** Real industrial AMD composition.

Constituencies	Concentration (mg/L)	Std Dev
Ca^2+^	483	2.65
Fe^2+^	895	4.52
Mg^2+^	308	3.95
Mn^2+^	195	4.89
Na^+^	153	1.38
Ni^+^	2.8	1.57
SO_4_^2−^	3680	3.28

The crossflow was first flushed with soapy water to get rid of any dirt trapped inside which could interfere with accuracy and consistency of the system. Thereafter, deionized water was used to thoroughly rinse the system before assembling the membranes in the cells having 125×75 mm dimensions (Area = 93.75 cm^2^). Pressure regulating valve having maximum value of 1000 psi is fitted downstream the feeding pump and was used to control pressure. The pressure controlling valve was used to set the desired pressure by throttling the pump discharge. The membranes were compacted at 7 bars for 4 hours using deionized water. Firstly, pure water flux (using Eq. 4) was determined by varying the pressure from 0.7 to 6.9 bars, to establish the original properties of the membrane. Then, AMD solution was filtered through to examine the rejection efficiency of the membranes through the crossflow system at different pressure. Flux of the AMD solution was also determined and compared to that of pure water flux. Rejection of the membranes was determined using Eq. (5). The system was operated at room temperature measured as 26 °C on the day.

To investigate the antifouling properties and operational stability of the membranes, pure water and industrial AMD were rapidly added to the feed tank and filtered through the membranes. First pure water flux was measured every 30 minutes for 6 hours at 6.9 bar based on the permeate volume collected. The pure water flux after 6 hours was measured as *J*_*w*1_ Thereafter, AMD solution was filtered, and similar exercise was conducted to establish AMD flux (*J*_*AMD*_) after 6 hours. After AMD was permeated through, pure water was filtered through the membrane again for 6 hours at 6.9 bars to establish any flux (*J*_*w*1_) loss. Backwashing was conducted and new pure water flux (*J*_*w*2_) was measured again for 6 hours at 6.9 bars.

**Figure 15:**
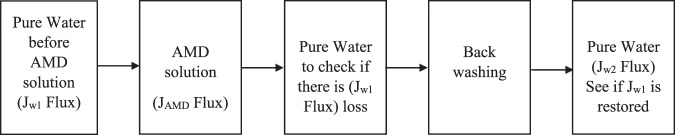
Experimental procedure for understanding membrane fouling and recovery of membrane flux.

Antifouling and operational stability of the optimized membrane was investigated using real industrial membrane in the crossflow filtration module. For comparison, the Flux Recovery Ratio (FRR), the Reversible Resistance (R_r_) and Irreversible Resistance (R_ir_) of the optimized PES/chitosan/PA membrane were determined using Eqs (6–8), respectively.

6 $$FRR=(\frac{{J}_{w2}}{{J}_{w1}})\times 100$$

7 $${R}_{r} \% =(\frac{{J}_{w2}-{J}_{AMD}}{{J}_{w1}})\times 100$$

8 $${R}_{ir} \% =(\frac{{J}_{w1}-{J}_{w2}}{{J}_{w1}})\times 100$$

where *J*_*w*1_, the water flux (l/m^2^h) was calculated using Eq. (4) and after that, flux (*J*_*AMD*_) of AMD feed (foulant solution) was measured and water flux was measured again to determine if there was loss of flux to warrant backwashing. Furthermore, the membrane was cleaned with deionized water and the permeate of cleaned membrane was measured again as *J*_*w*2_ (l/m^2^h) to confirm restoration of original flux to substantiate the effect of backwashing. Figure 15 depicts step by step procedure which was followed to conduct fouling experiments. Fouling was circumvented by hydraulic cleaning (backwashing) which is a reversed filtration process whereby water is permeated in the opposite direction to expand the fouling layer and fluidises it for ease of removal of trapped contaminants. The flux measurements were taken after almost steady state is reached and replicate measurements were taken, and average values reported.
